# Investigating the Impact of AI-Supported Self-Coaching as a Professional Development Model for Embedded Instruction in Inclusive Early Childhood Settings

**DOI:** 10.3390/bs16010140

**Published:** 2026-01-19

**Authors:** Serife Balikci

**Affiliations:** 1 Early Childhood Education/Teacher Preparation Program, Piedmont Community College, Roxboro, NC 27574, USA; srakap86@piedmontcc.edu; Tel.: +1-336-322-2174; 2 Carsamba District Directorate of National Education, 55500 Samsun, Türkiye

**Keywords:** artificial intelligence (AI), self-coaching, embedded instruction (EI), inclusive early childhood education, autism spectrum disorder, preschool teachers

## Abstract

This study examined the effectiveness of an Artificial Intelligence (AI)-supported self-coaching system designed to improve preschool teachers’ implementation of embedded instruction (EI) for young children with autism in inclusive early childhood classrooms. Using a multiple-probe across participants single-case design with four teacher–child dyads, the study evaluated changes in teacher fidelity, child learning outcomes, maintenance, generalization, and teacher perceptions. Following baseline and an initial EI training, teachers engaged in weekly AI-supported self-coaching cycles that included planning, data entry, reflection, and AI-generated individualized feedback. Results demonstrated clear functional relations between the introduction of the AI-supported system and increases in teachers’ EI fidelity. All teachers reached high levels of accurate implementation, maintained their performance after AI supports were withdrawn, and generalized EI procedures to non-targeted routines. Correspondingly, children showed substantial improvements in unprompted correct responding on individualized goals, with gains sustained across maintenance and generalization probes. Social validity data indicated that teachers found both EI and AI-supported self-coaching highly acceptable, feasible, and helpful for guiding instructional decision-making. Findings provide promising initial evidence that AI-supported self-coaching can serve as a scalable, cost-effective professional development approach that strengthens teacher practice and enhances learning outcomes for young children with autism in inclusive preschool settings.

## 1. Introduction

Inclusive early childhood education environments provide essential opportunities for young children with autism to develop cognitive, social, and communication skills alongside their typically developing peers (Balikci et al., 2025a). When children participate in everyday routines, activities, and transitions with appropriate supports, they are more likely to demonstrate meaningful engagement and behavioral regulation and acquire new skills that generalize across settings and persist over time (Lawrence et al., 2016; Odom et al., 2011). However, the benefits of inclusive placement are not realized automatically. For inclusion to function as a meaningful opportunity rather than simply a physical placement, children must be supported by instructional strategies that are individualized, intentional, and delivered within the ongoing flow of classroom life (Lawrence et al., 2016; Odom et al., 2011; Rule et al., 1998). Without such instructional supports, inclusive environments may risk replicating patterns of participation inequality, where children with disabilities are physically present but not engaged or learning at rates comparable to their peers (Balikci et al., 2025b).

Naturalistic instructional approaches have emerged as a cornerstone of high-quality inclusive early childhood practice because they embed learning opportunities within authentic contexts (Snyder et al., 2015). These approaches provide instruction during play, caregiving routines, and classroom transitions rather than isolated, adult-directed sessions. Research consistently shows that when children with autism learn in functional and purposeful contexts, they demonstrate higher motivation, greater task participation, and more robust generalization compared to decontextualized instruction (Daugherty et al., 2001; Rakap, 2013; Snyder et al., 2015). Naturalistic instruction also aligns with the principles of developmental appropriateness and child-centered pedagogy, which prioritize play, interest-responsive teaching, and active participation (Rakap, 2017b).

Embedded instruction (EI) is one of the most empirically supported naturalistic instructional approaches used in inclusive preschool settings (Gulboy et al., 2023; Rakap & Gülboy, 2024; Rakap & Parlak-Rakap, 2011; Snyder et al., 2015). It involves distributing brief, intentional learning trials throughout daily events, each targeting an individualized priority learning objective identified through assessment and individualized education program (IEP) planning (Snyder, 2025). When implemented with fidelity, EI enables children to participate fully in the classroom while simultaneously receiving systematic learning opportunities to advance communication, social interaction, cognitive development, and pre-academic skills (Rakap & Parlak-Rakap, 2011; Snyder et al., 2015). The approach is inherently individualized, allowing teachers to align instruction with a child’s strengths, interests, and developmental needs while maintaining the natural flow of daily routines.

The evidence base supporting EI is extensive and compelling. A recent meta-analysis confirmed that EI meets criteria for an evidence-based practice, demonstrating large effects across multiple developmental domains and generalization and maintenance of skills across contexts and time (Gulboy et al., 2023). Furthermore, EI strengthens not only skill acquisition but also participation and belonging by enabling children with and without disabilities to engage meaningfully in shared classroom experiences (Frazeur Cross et al., 2004; Jimenez & Kamei, 2015; Rakap et al., 2024). Recent empirical studies have added particularly important insights. Rakap et al. (2024) showed that embedded learning opportunities promote individualized support and meaningful participation for all children by integrating instruction seamlessly into daily activities. Teachers in that study showed only modest improvement in EI performance following workshop-style PD, but they achieved and sustained criterion-level fidelity only after receiving practice-based coaching (PBC). Child learning outcomes improved in parallel with increases in teacher fidelity. Similarly, Balikci et al. (2025a) demonstrated that embedded learning opportunities not only enhance skill development but also significantly increase engagement for preschool-aged children with autism and intellectual disability. Children maintained and generalized their engagement across settings and made progress on their targeted learning objectives, and teachers rated both the practices and the PD model as socially valid. Together, the most current research reinforces two critical points: EI produces powerful developmental benefits, and high-fidelity use of EI is most reliably achieved and sustained when teachers receive ongoing implementation support, particularly coaching.

Despite decades of empirical support, EI continues to be implemented inconsistently in authentic early childhood classrooms. Many teachers report learning about EI during preservice preparation or professional development, yet they struggle to translate conceptual understanding into sustained high-quality implementation in daily practice (Balikci et al., 2025a; Noh et al., 2009; Rahn et al., 2019). Recent observational evidence confirms this implementation gap. In a study of 41 preschool teachers, Balikci et al. (2025b) found that teachers used embedded learning opportunities infrequently and inconsistently across observations despite working in inclusive classrooms with children who had individualized goals. Although teachers were more likely to address language and cognitive goals and tended to embed instruction during transitions, routines, or child-directed activities, large variation was observed both between and within teachers across observation sessions. Furthermore, teacher-level variables (e.g., experience with children with disabilities, total years of experience, number of children or adults in the classroom) did not predict teachers’ use of EI. Collectively, these findings highlight that even when teachers possess foundational knowledge of EI, the demands and complexity of natural classroom environments make it difficult to plan, deliver, monitor, and adapt EI without systematic implementation support. As a result, children with autism may receive few or inconsistent instructional trials, limiting the developmental benefits that EI can offer.

A large body of literature identifies professional development as a critical driver of teachers’ sustained and accurate use of evidence-based practices such as EI. PD models that pair initial training with ongoing implementation support, especially coaching, produce the most reliable and lasting changes in teacher behavior (Kretlow & Bartholomew, 2010; Snyder et al., 2018). For example, the Tools for Teachers intervention demonstrated that teachers who received on-site coaching implemented EI more frequently and with higher fidelity than those who received training alone or those in the business-as-usual condition, and their children exhibited greater developmental progress (Snyder et al., 2018). Similarly, Rakap (2017a) found that preservice teachers demonstrated high-fidelity EI only after receiving individualized live coaching, and gains were sustained and generalized across routines over time. Other research has replicated these findings across cultural contexts and PD delivery formats, including e-coaching and telepractice supports, underscoring that coaching is a key mechanism for supporting and maintaining implementation fidelity (Ai et al., 2024; Coogle et al., 2017; Rakap et al., 2024).

Despite the clear benefits of coaching-based PD models, they remain resource-intensive. In-person coaching demands substantial allocations of personnel time, travel, scheduling coordination, and specialized training, limiting feasibility for many early learning programs (Cilliers et al., 2022; Schachner et al., 2024). Self-coaching where teachers independently analyze implementation data and reflect on their teaching has been proposed as an alternative, but self-coaching requires specialized tools and strong intrinsic motivation and typically lacks the individualized feedback that is central to performance improvement (Shannon et al., 2015). Thus, the field continues to face a persistent challenge: how to make high-quality PD and coaching accessible and scalable so that all early childhood educators, not just a subset working in well-resourced programs, can implement evidence-based practices like EI (Powell & Diamond, 2019; Sheridan et al., 2009).

Rapid advances in artificial intelligence (AI) present a promising solution to this challenge. AI-supported self-coaching systems are emerging as novel PD tools that can generate personalized, data-driven feedback and guide teacher decision-making without requiring continuous involvement of a live coach (Rakap, 2024). By automating core coaching functions such as analyzing performance data, identifying implementation needs, and suggesting action steps, AI-supported self-coaching has the potential to maintain the learning benefits of coaching while dramatically reducing cost and personnel demands. For early childhood programs that struggle to provide ongoing coaching due to geographic, staffing, or financial constraints, AI-supported self-coaching may represent an especially powerful and equitable alternative.

Recent advances in AI have created new opportunities to extend established professional development models by automating select components of feedback and reflection while preserving teacher agency. AI-supported self-coaching builds conceptually on prior work in self-coaching and practice-based coaching by offering structured, data-driven guidance without requiring continuous access to a live coach. Importantly, the present study conceptualizes AI not as a replacement for professional expertise, but as a tool designed to support teachers’ reflective practice within well-established, evidence-based instructional frameworks.

However, research on AI-enabled professional development in early childhood special education is extremely limited, and no studies have examined whether AI-supported self-coaching can improve teacher implementation of EI or enhance child developmental outcomes. The field currently lacks empirical evidence regarding whether AI-based systems can (a) promote improvements in teacher use of evidence-based practices, (b) support children’s progress toward individualized goals, and (c) be perceived by teachers as feasible and supportive of professional growth. Addressing these gaps is necessary to determine whether AI-supported coaching can advance the goals of high-quality, inclusive education while easing the infrastructural burden associated with traditional coaching.

The present study responds to this need by developing and evaluating an AI-supported self-coaching system designed specifically to help preschool teachers implement EI for young children with autism in inclusive settings. The system integrates evidence-based EI procedures with AI-generated individualized feedback to support teachers in applying the practice independently in authentic classroom contexts. The study builds on decades of research documenting the importance of coaching in promoting sustained practice use while addressing longstanding challenges related to scalability, cost, and accessibility.

Guided by this purpose and the gaps in the literature, the present study addressed the following research questions:

1. Does AI-supported self-coaching improve preschool teachers’ instructional fidelity and use of embedded instruction learning trials across daily classroom routines?
2. To what extent do teachers generalize and maintain the use of embedded instruction learning trials after the completion of AI-supported self-coaching?
3. What is the impact of improved teacher instructional fidelity on the developmental and learning outcomes of autistic preschoolers, with particular emphasis on progress toward IEP goals?
4. How do preschool teachers perceive the usability, acceptability, feasibility, and impact of AI-supported self-coaching on their instructional practice and professional growth?

## 2. Method

The study received approval from the university’s Institutional Review Board, and all research procedures complied with ethical standards for work with young children and educators. Necessary permissions were obtained from participating early childhood programs prior to recruitment. Parents of all child participants were fully informed about study activities and provided written consent, and teachers provided written consent for their own participation. To protect confidentiality, identifiable information was removed from all data sources and reports.

### 2.1. Experimental Design

A multiple-probe across participants single-case experimental design (Ledford & Gast, 2018) was used to examine the effects of a professional development intervention consisting of (a) training on EI and (b) AI-supported self-coaching (independent variable) on preschool teachers’ fidelity of EI implementation and associated learning outcomes for young children with autism (dependent variables). This design allows systematic evaluation of functional relations between the introduction of the intervention and subsequent changes in teacher and child behavior while controlling for threats to internal validity through staggered introduction of the independent variable. The multiple-probe variation was specifically selected because EI trials occur naturally within daily classroom routines rather than in massed, continuous formats. Thus, continuous baseline measurement was neither feasible nor necessary to establish experimental control.

The design included four phases: baseline, training, AI-supported self-coaching, and maintenance. Baseline sessions were collected concurrently across all dyads until stability or a descending trend was observed. Training was introduced to all teachers simultaneously; however, AI-supported self-coaching was introduced in a staggered manner across teachers to permit demonstration of a functional relation. Only one teacher at a time entered the AI-supported self-coaching phase, while others remained in the post-training condition to rule out maturation, history, and exposure to training alone as plausible explanations for changes in performance. Teachers remained in each phase until meeting pre-specified criteria for stability before transitioning. The maintenance phase was used to assess the durability of the intervention effect and to evaluate teacher generalization of EI procedures in the absence of AI support.

### 2.2. Participants

Four teacher–child dyads participated in the study. Each dyad consisted of one preschool teacher and one child with autism who was enrolled in that teacher’s inclusive early childhood classroom. Teachers were recruited from three community-based early learning settings. Recruitment occurred through outreach to program administrators, informational emails, and follow-up conversations with teachers who expressed interest. All participants met eligibility criteria and completed informed consent procedures prior to participation. To be eligible, teachers were required to (a) be responsible for instruction in a classroom serving children ages 3–5, (b) work in a program that enrolled children with and without disabilities together, (c) have at least one year of teaching experience, (d) currently serve a child with a documented diagnosis of autism spectrum disorder, and (e) agree to participate in training, structured AI-supported self-coaching sessions, and video-based data collection. Teachers did not receive financial compensation or additional incentives for participation beyond access to professional development, training materials, and the AI-supported self-coaching system.

Children were eligible for inclusion if they (a) had a documented ASD diagnosis, (b) were between 3 and 5 years old, (c) had an active IEP that included measurable instructional objectives, and (d) attended school for at least 80% of scheduled instructional hours. After parental consent was obtained, one child meeting eligibility criteria was selected from each classroom. Children displayed diverse developmental profiles, typical of preschoolers with ASD enrolled in inclusive early childhood programs.

#### 2.2.1. Dyad 1 (Teacher 1—Child 1)

Teacher 1 was a female educator aged 28 years, with a bachelor’s degree in early childhood education and 5 years of teaching experience. She taught in a mixed-age inclusive preschool classroom with 17 children, including two with identified disabilities. She reported familiarity with developmental screening tools and general early childhood instructional strategies but limited prior experience implementing EI within naturally occurring routines. Child 1 was a 48-month-old boy with a documented diagnosis of ASD. He communicated using a combination of gestures, vocalizations, and emerging single-word approximations. He demonstrated strengths in visual attention, persistence with preferred materials, and exploratory play; however, he experienced challenges initiating communication, following multi-step verbal directions, and engaging with peers during play. His ABILITIES Index profile indicated mild-to-moderate limitations in intentional communication, social interaction, and motor coordination. His IEP goals targeted expressive language, symbolic play, and responding to adult prompts during routines. He received weekly speech-language therapy as part of his preschool program.

#### 2.2.2. Dyad 2 (Teacher 2—Child 2)

Teacher 2 was a female educator aged 33 years, with a bachelor’s degree in early childhood education and 9 years of classroom experience, including work in bilingual early childhood settings. Her classroom served 15 children and emphasized family engagement and bilingual communication. She had experience with general behavioral supports but no formal preparation in EI or naturalistic teaching approaches. Child 2 was a 58-month-old bilingual girl with ASD. She demonstrated strong fine motor abilities and interest in structured, table-based activities, but had difficulty with flexible thinking, shifting attention, and responding appropriately to social bids. Her expressive language consisted primarily of single words and familiar phrases in both languages. Her ABILITIES Index scores reflected moderate limitations in social communication, attention regulation, and adaptation to transitions. Her IEP goals focused on increasing joint attention, learning early academic concepts, and engaging more independently during small-group activities. She received speech-language therapy and participated in a bilingual literacy enrichment group.

#### 2.2.3. Dyad 3 (Teacher 3—Child 3)

Teacher 3 was a female educator, aged 40 years, with a bachelor’s degree in child development and 14 years of experience in early care and education. Her full-day preschool classroom served 18 children and followed a structured schedule that incorporated both teacher-led instruction and child-directed exploration. Although she expressed confidence in classroom management and routine-based instruction, she had limited experience planning individualized learning opportunities within ongoing activities. Child 3 was a 52-month-old boy with ASD. He demonstrated emerging receptive language and was able to imitate familiar motor actions, but had difficulty sustaining engagement, initiating communication, and maintaining attention during peer play. His ABILITIES Index profile indicated moderate limitations in intentional communication, social reciprocity, and adaptive functioning. He responded well to visual supports and predictable routines yet required adult prompting and scaffolding to complete multi-step tasks. His IEP goals targeted functional communication, participation in group routines, and increasing independent responding during structured and play-based instruction. He received school-based speech-language and occupational therapy.

#### 2.2.4. Dyad 4 (Teacher 4—Child 4)

Teacher 4 was a female educator, aged 34 years, with a master’s degree in early childhood special education and 7 years of experience teaching in blended inclusive preschool programs serving both children with and without disabilities. Her classroom included 16 children and followed a consistent daily routine that incorporated small-group learning, center-based activities, and outdoor play. She had prior experience receiving performance feedback through coaching but had not previously used an AI-supported self-coaching model. Child 4 was a 44-month-old girl with ASD. She demonstrated strong visual-spatial reasoning and emerging problem-solving skills but experienced challenges with adaptive behavior, emotional regulation, and reciprocal social interaction. She communicated using short phrases supplemented by visual supports but struggled with conversational reciprocity and peer engagement. Her ABILITIES Index profile indicated mild-to-moderate limitations in social communication, behavioral regulation, and functional independence. Her IEP goals emphasized cooperative play, following multi-step routines with fewer prompts, and expanding expressive language. She received weekly speech-language therapy and classroom-embedded social–emotional supports.

### 2.3. Settings and Materials

The study was conducted within the participating teachers’ inclusive preschool classrooms during their regularly occurring daily routines. Classrooms were located in suburban and rural communities in the southeastern region of the country and served between 15 and 18 children. Each environment was equipped with typical early childhood materials including blocks, dramatic play props, art supplies, manipulatives, sensory bins, and book areas that supported a wide range of instructional and play opportunities. Teachers also used a variety of visual supports, such as picture schedules, first–then boards, and token systems, depending on the needs of their students. No special equipment or instructional materials were introduced by the research team, as the use of familiar, naturally occurring routines aligns with the foundational principles of EI.

Teacher training for the study was conducted remotely using the Zoom videoconferencing platform. All teachers participated in a synchronous, researcher-led training session featuring live instruction, screen-shared slide presentations, video models of EI, and guided practice with application activities. Digital training materials including EI guides, planning templates, and sample instructional scenarios were distributed electronically prior to the session. Breakout rooms, chat features, and annotation tools were used to facilitate participation and provide opportunities for teachers to apply concepts collaboratively.

Individualized training on the AI-supported self-coaching system also took place through Zoom. Each teacher participated in a one-on-one session during which a research team member modeled how to log in to the platform, enter de-identified instructional and child performance data, interpret graphical feedback, and respond to weekly reflection prompts. Teachers practiced completing each step using screen-shared examples and received immediate support for any questions or troubleshooting needs.

The AI-supported self-coaching system was accessed through a secure, password-protected website designed to guide teachers through the full cycle of EI planning, implementation, and reflection. Teachers used personal laptops, Chromebooks, or school-issued tablets to access the system. The platform provided structured prompts for identifying instructional objectives, selecting routines for EI, documenting implementation accuracy and child responses, and reflecting on the week’s instructional strengths and needs. AI-generated outputs included graphical displays of teacher fidelity trends and child performance data, as well as narrative feedback offering individualized recommendations for upcoming instructional cycles. All data were stored securely in the system and were accessible only to participating teachers and authorized members of the research team.

### 2.4. Study Procedures

#### 2.4.1. Pre-Baseline

Selection of the AI Tool. Before any classroom data were collected, the research team completed a systematic process to select the AI tool for self-coaching used in the intervention. First, unpaid versions of the following AI platforms ChatGPT 4, Gemini 1.5 and Microsoft Copilot (https://copilot.microsoft.com/) were evaluated for their capacity to (a) support structured prompting sequences, (b) accept and process teacher-entered data, and (c) generate individualized narrative feedback aligned with EI procedures. Selection was guided by considerations of accessibility, user-friendliness, reliability, and compliance with ethical guidelines relevant to early childhood and disability-focused educational research. To strengthen the evaluation process, two experienced early childhood teachers were invited to participate in trialing and rating each platform. These teachers provided feedback on clarity of responses, usability within a preschool workflow, and the extent to which each platform produced feedback that felt supportive, accurate, and developmentally appropriate. Their insights were incorporated into the final selection process. Based on the combined assessment of technical capabilities, ethical considerations, and practitioner feedback, an open-domain version of ChatGPT 4 was selected as the AI platform used during the AI-supported self-coaching phase. The AI system did not operate autonomously or as a closed instructional decision-making system. Instead, it functioned as a structured, open-domain generative tool guided entirely by researcher-developed prompt templates grounded in established EI and practice-based coaching frameworks.

Development of the AI-Supported Self-Coaching Protocol. The development of the self-coaching protocol followed an iterative, user-centered design process. The research team created a structured prompting framework organized around both the four procedural components of EI (i.e., what to teach, when to teach, how to teach, and how to evaluate; Snyder, 2025) and the core phases of PBC (Rakap et al., 2025; Snyder et al., 2022): goal setting/planning, implementation/data collection, and reflection with feedback. Prompting sequences were intentionally fixed and linear to ensure consistency across teachers and weeks, with each coaching cycle requiring teachers to complete all stages before advancing to the next.

For each procedural element, the team created detailed prompt templates and logic chains designed to guide teachers through (a) identifying instructional goals; (b) selecting routines in which EI trials would occur; (c) specifying antecedents, prompts, reinforcement, and error-correction procedures; (d) documenting trial accuracy; and (e) interpreting patterns in child learning. Teachers entered structured, short-response information (e.g., number of trials delivered, child response types, contextual barriers), which limited open-ended input and reduced the likelihood of irrelevant or non–evidence-based AI output.

All AI-generated feedback was constrained by researcher-developed prompt rules that explicitly restricted responses to evidence-based EI components and practice-based coaching principles. The system did not generate new instructional strategies; rather, it synthesized teacher-entered data and reflected it back through structured summaries, graphical displays, and targeted reflective questions (e.g., identifying missed antecedents or inconsistent consequence delivery). This design ensured that the AI functioned as a guided self-reflection and feedback tool rather than as an autonomous instructional decision-maker.

To refine usability, the research team partnered with three experienced preschool teachers who had prior experience implementing EI with children with autism. These teachers reviewed and piloted the initial prototype, completing simulated coaching cycles using mock data and responding to structured reflection prompts. Usability sessions focused on clarity of prompt language, appropriateness of task sequencing, feasibility within classroom schedules, and accuracy of AI-generated recommendations. Feedback from these sessions resulted in revisions to prompt organization, the phrasing of reflection questions, examples used within the tool, and formatting of the AI’s graphical and narrative outputs.

Ethical Safeguards. Ethical and privacy safeguards were integrated throughout protocol development. Teachers were formally trained to remove all identifying information (child names, initials, dates of birth, program names, etc.) before entering data into the AI system. Training explicitly addressed the limitations of generative AI, including the potential for bias, overgeneralization, or hallucinated content. Teachers were informed that AI-generated feedback was advisory in nature and that all instructional decisions required professional judgment and alignment with evidence-based EI procedures. To minimize the risk of biased or non-evidence-based recommendations, all AI prompts were pre-structured by the research team and constrained to EI components supported in the empirical literature (e.g., antecedents, response intervals, reinforcement, and error correction). The research team monitored AI-generated outputs during the study to ensure consistency with EI principles and ethical instructional practices. Teachers were also encouraged to flag any AI-generated feedback they perceived as unclear or inappropriate, although no such incidents were reported. Teachers were also provided explicit instructions regarding data security and confidentiality.

Descriptive Data Collection. Following consent from teachers and parents, descriptive data were collected to characterize the context of the study. Each participating teacher completed a demographic questionnaire that included items about their educational background, years of experience, classroom size, professional development history, and familiarity with naturalistic instructional strategies. Teachers also completed the ABILITIES Index (Simeonsson & Bailey, 1991) to profile the functional strengths and needs of the participating child across nine developmental domains. The index rates functioning in areas such as communication, social behavior, motor abilities, sensory functioning, and overall health were determined using a six-point scale, with higher scores indicating greater levels of support need. The ABILITIES Index has demonstrated strong psychometric properties, including acceptable reliability and evidence of construct validity for describing functional abilities in young children with disabilities (Buysse et al., 1993). These data were used only for descriptive purposes and to guide the selection of appropriate target behaviors.

Identifying Instructional Objectives. Prior to the start of baseline, each teacher identified three instructional objectives for their participating child. Objectives could be taken directly from the child’s IEP, adapted from existing goals, or newly developed based on teacher understanding of the child’s developmental needs. Teachers were encouraged to select goals across multiple developmental domains (e.g., communication, social interaction, early cognitive skills) in alignment with EI. The chosen instructional objectives served as the basis for identifying when EI trials occurred, determining the accuracy of teacher implementation, and evaluating child learning outcomes throughout the study. Table 1 presents the instructional objectives identified for each child, the routines in which they were targeted, and sample antecedent–behavior–consequence sequences illustrating how EI trials were embedded within daily activities.

#### 2.4.2. Baseline

During the baseline phase, teachers were instructed to continue their typical teaching practices without receiving any training, guidance, or support related to EI or AI-supported self-coaching. Baseline was used to document naturally occurring levels of teacher implementation fidelity and child performance on their targeted instructional objectives.

Each teacher–child dyad participated in six baseline sessions. Observers recorded all instructional trials that teachers naturally delivered during typical classroom routines (see Table 1), noting whether antecedents, responses, and consequences were implemented correctly. No feedback was provided to teachers during this phase. Data from the baseline phase allowed the research team to determine the stability of teacher and child performance prior to intervention and to establish a comparison point for evaluating the effects of training and AI-supported self-coaching.

**Table 1:** Instructional Objectives, Routines, and Embedded Instruction Examples.

Child	Instructional Objective	Routine—Intervention/ Generalization	Antecedent	Behavior	Consequence
Child 1	Use 1–2-word phrases to request preferred items	Centers/ Table Activities	Teacher holds snacks and asks, ‘What do you want?’	Child says, ‘Crackers please.’	Teacher gives the crackers, labels the child’s request (“You said crackers, great asking!”).
	Follow simple 2-step directions	Transitions/ Snack	Teacher says, ‘Put the car in the bin and sit on the carpet.’	Child completes both steps.	Teacher offers specific praise (“You put the car away and sat on the carpet-nice listening!”).
	Label familiar objects	Centers/Table Activities	Teacher points to a picture and asks, “What is this?”	Child names the object.	Teacher provides praise and expands: “Yes, apple! A red apple.”
Child 2	Shift attention to an item the teacher points to	Circle Time/Centers	Teacher points to picture and says ‘Look!’	Child looks at object then teacher.	Teacher praises: “Nice looking!” and labels the item.
	Name basic shapes (circle, square, triangle)	Table Activities/ Centers	Teacher holds up a shape card and asks, “What shape is this?”	Child names the shape.	Teacher provides confirmation and expansion: “Yes, triangle-three sides!”
	Complete a simple fine-motor task independently	Small Groups/ Centers	Teacher places materials and gives direction: “Match each picture to the same one.”	Child completes the task with no more than one prompt.	Teacher reinforces: “Great matching!” and provides next step/activity.
Child 3	Use 1–2-word phrases to request help	Centers/Snack	Teacher withholds item and asks, ‘What do you need?’	Child says ‘Help’ or ‘Open.’	Teacher gives the needed help, labels the child’s request (“Nice asking for help!”).
	Label basic colors (red, yellow, blue)	Centers/ Snack	Teacher holds up a colored object (e.g., block, crayon) and asks, “What color is this?” while pointing to the item.	Child says “red,” “yellow,” or “blue.”	Teacher provides immediate confirmation and expansion (e.g., “Yes, blue! You found the blue block.”) and allows child to continue the activity.
	Follow a one-step transition direction	Transitions/ Snack	Teacher gives a clear direction (e.g., “Time to clean up-put toys in the basket.”) with a gesture toward the materials.	Child follows the direction independently or with one prompt.	Teacher gives specific praise: “Great cleaning up-thank you!”
Child 4	Use 1–2-word social phrases (e.g., “my turn,” “play?” “help”)	Centers/ Snack	Teacher approaches child and prompts, “*What do you want to say?*” or “*Your turn to ask*.”	Child uses a short phrase such as “my turn,” “play?” or “help.”	Teacher responds immediately (e.g., gives the turn, joins play, or provides assistance) and models the next phrase.
	Follow a 2-step routine	Transitions/ Snack	Teacher gives a clear 2-step direction (e.g., “*Get your cup, then sit on the carpet.*”).	Child completes both steps in order (gets object, moves to location).	Teacher provides labeled praise (e.g., “*You followed both steps-great job!*”) and continues transition.
	Take 2–3 cooperative play turns	Centers/Table Activities	Teacher sets up a turn-taking activity and models the first turn, saying “*My turn, now your turn.*”.	Child takes 2–3 turns by rolling ball back, adding block, or passing toy.	Teacher provides enthusiastic praise, expands play, and keeps the interaction going for additional turns.

#### 2.4.3. Training

Embedded Instruction. Following the completion of baseline data collection, all four teachers participated in a professional development program designed to prepare them to implement EI with fidelity. The training was delivered synchronously via Zoom and consisted of a single, intensive 4 h session focused on the foundational principles, procedures, and applications of EI. The content and organization of the training were informed by the empirical literature on naturalistic instructional practices and embedded learning opportunities and adapted from Rakap et al. (2024). Consistent with recommendations that PD incorporate the core components of planning, instruction, and assessment, the training session systematically introduced teachers to the four procedural elements of EI, i.e., what to teach, when to teach, how to teach, and how to evaluate child learning.

The session included explicit instruction accompanied by video exemplars that illustrated both correct and incorrect implementation of EI within real preschool routines. Teachers practiced identifying appropriate target behaviors, selecting natural routines for embedding instruction, and analyzing instructional scenarios to determine whether complete learning trials had occurred. Breakout room activities, annotation tools, and guided practice ensured active engagement throughout the session. Teachers also worked through sample instructional planning templates that aligned their selected child objectives with specific routines, antecedent strategies, and consequence procedures. These planning tasks helped teachers begin envisioning the integration of EI within their everyday classroom activities, which is a critical component of transferring training content into practice.

Digital training materials including an EI manual, planning templates, and sample trial scripts were provided to teachers prior to the session to support both immediate practice and later reference during implementation. Throughout the training, instructors facilitated discussions about the practical challenges of embedding instruction in busy classroom contexts, strategies for maintaining natural flow while delivering systematic instruction, and ways to ensure alignment between EI and children’s individualized learning goals. These reflective conversations were consistent with recommended PD practices that emphasize collaborative learning, problem-solving, and contextualized application.

AI-Supported Self-Coaching. After completing the EI training and before moving into the AI-supported self-coaching phase, each teacher participated in a separate individualized two-hour training session focused specifically on using the AI-supported self-coaching platform. Delivered one-on-one via Zoom, these sessions familiarized teachers with the structure, purpose, and weekly expectations of the AI self-coaching cycle. Teachers practiced logging into the secure platform, entering de-identified instructional data, recording child performance outcomes, and responding to the structured reflection prompts that would guide weekly self-evaluation. Research team members used screen sharing to demonstrate each function of the system and provided immediate support as teachers practiced entering mock data and reviewing sample AI-generated outputs. Teachers learned to interpret graphical displays of their implementation fidelity and child performance and to use the AI-generated narrative recommendations to guide their planning for subsequent weeks. These individualized sessions ensured that teachers demonstrated proficiency with the platform and understood how to use the system to support accurate, reflective, and data-informed implementation of EI before entering the intervention phase.

#### 2.4.4. Post-Training

Immediately following the completion of the EI training and prior to initiating the AI-supported self-coaching phase, all four teachers were asked to implement EI independently, using only the knowledge and materials provided during training. This post-training period served to evaluate the extent to which training alone influenced teacher implementation and child performance. During this phase, teachers were encouraged to apply the EI procedures they had learned (identifying appropriate routines, delivering complete instructional trials, and responding to child behavior using systematic consequence strategies) but they did not receive any additional coaching, guidance, or feedback from the research team. After six post-training sessions had been completed, the teacher whose data showed the most stable pattern of performance was selected to transition into the AI-supported self-coaching phase. Post-training data collection continued intermittently for the remaining teachers until each entered the AI-supported self-coaching phase.

#### 2.4.5. AI-Supported Self-Coaching

The AI-supported self-coaching phase functioned as an automated, teacher-led adaptation of PBC (Balikci et al., 2025c; Rakap et al., 2025; Snyder et al., 2022), incorporating the core components of goal setting, observation, and reflection/feedback through an AI-mediated process rather than through a live coach. Once a teacher entered this phase, she began engaging in weekly coaching cycles consisting of (a) structured planning, (b) implementation and data collection within ongoing classroom routines, and (c) reflection supported by individualized AI-generated feedback. To support secure and consistent use of the AI system, the research team created individual ChatGPT 4 accounts for each teacher, provided secure login credentials, and monitored teacher engagement weekly (e.g., login frequency, completeness of data entry, responsiveness to prompts).

During the planning phase, teachers reviewed their target instructional objectives and identified the specific routines in which EI would occur. The AI platform guided teachers through a sequenced set of prompts designed to help them articulate what to teach, when to teach, and how to teach, including specifying antecedents, prompting procedures, reinforcement strategies, and planned error-correction actions. This structured planning process supported intentional and systematic implementation of EI and ensured that teachers’ decisions were directly aligned with children’s individualized learning needs and reflecting the goal-setting and action-planning components characteristic of PBC.

During the implementation and data collection phase, teachers used EI within naturally occurring routines across the week and documented their instructional delivery. Teachers entered the number of trials delivered, the accuracy with which they completed essential EI components (i.e., antecedent, prompt, response interval, consequence), and the child’s response type (unprompted correct, prompted correct, incorrect, or no response) into AI platform. Teachers were expected to enter data at least once per week, though several chose to enter data more frequently depending on classroom schedules and opportunities for EI implementation. The research team’s monitoring of platform use allowed for verification that teachers were engaging with the tool consistently and entering sufficient instructional data for the AI to generate meaningful feedback.

During the reflection and AI-generated feedback phase, teachers logged into the platform at the end of each week, typically on Fridays, to respond to structured reflection prompts addressing successes, challenges, and contextual factors influencing implementation. The AI system synthesized teacher-entered data and produced individualized feedback that included graphical summaries of fidelity and child performance trends, narrative descriptions of strengths, and data-based recommendations for the subsequent instructional cycle. Feedback emphasized supportive and constructive guidance aligned with evidence-based instructional principles, similar in function to the reflective conversation and feedback meetings in PBC. The AI’s prompts encouraged teachers to consider needed adjustments, upcoming opportunities for embedding instruction, and environmental or instructional barriers that could be addressed in the next week. Teachers remained in the AI-supported self-coaching phase until they achieved at least 80% fidelity across three consecutive sessions or until 8 weeks had passed, whichever occurred first.

### 2.5. Maintenance

Once teachers met fidelity criteria during the AI-supported self-coaching phase, they entered the maintenance phase. During maintenance, teachers continued implementing EI during ongoing routines but no longer had access to AI feedback.

Teachers retained access to their initial EI training materials but were explicitly instructed not to log into the AI system. At least three maintenance data points were collected to assess whether teachers sustained high levels of implementation after withdrawing the AI-supported coaching tool. Child learning outcomes were also monitored during maintenance to assess continuation of progress.

### 2.6. Generalization

To evaluate whether teachers’ use of EI and children’s target responses transferred beyond the specific routines practiced during the primary intervention sessions, generalization probes were conducted intermittently throughout the study. For each teacher–child dyad, one routine that was not used during baseline, post-training, AI-supported self-coaching, or maintenance sessions was identified collaboratively with the teacher (see Table 1). During these probes, teachers were instructed to engage in their typical classroom practices without receiving any advance planning support or access to the AI platform. No feedback was provided following generalization probes. These sessions allowed the research team to determine whether improvements in teacher implementation and child performance extended to new routines/settings.

### 2.7. Data Collection and Analysis

Teacher and child performance data were collected throughout all phases of the study using the Embedded Teaching Coding Form (Rakap & Balikci, 2017). Observations occurred two times per week during baseline and the first six sessions of post-training, and one time per week during the AI-supported self-coaching phase. After the first six post-training sessions, the remaining teachers who had not yet entered the AI-supported condition were observed intermittently, approximately to monitor changes in teacher implementation without additional support. Generalization probes and maintenance observations were also conducted intermittently, following the same procedures used during baseline and intervention. All observations took place during naturally occurring classroom activities in which the teacher interacted with the target child. A trained observer collected data live for each session, applying the ETCF to document teachers’ implementation of EI trials and children’s responses to instructional opportunities.

For each observed instructional opportunity, the observer coded whether the teacher delivered a complete or incomplete EI trial. A trial was coded as *complete* when (a) an antecedent or task direction was presented, (b) an appropriate response interval was provided, and (c) a consequence followed the child’s behavior, either reinforcement for correct responding or an error-correction procedure following incorrect or absent responding. A trial was coded as *incomplete* when any of these required components were missing (e.g., no wait time, omission of reinforcement or error correction).

Child performance was coded using four mutually exclusive categories: unprompted correct response, prompted correct response, incorrect response, or no response. An *unprompted correct response* was recorded when the child performed the target behavior independently within three seconds of the teacher-delivered antecedent. A *prompted correct response* was coded when the child responded correctly only after receiving a teacher-delivered prompt. An *incorrect response* indicated that the child engaged in a behavior other than the target skill, whereas *no response* indicated that the child did not demonstrate any observable behavior following the antecedent (and prompt, when applicable). For each session, the observer calculated (a) the percentage of complete EI trials implemented by the teacher and (b) the percentage of unprompted correct responses demonstrated by the child. These data were graphed immediately following each observation to allow for ongoing monitoring of fidelity and child performance across phases. Teacher implementation data served as the primary dependent variable for determining phase changes.

Data collected during baseline, post-training, AI-supported self-coaching, generalization, and maintenance phases were analyzed using visual analysis procedures. Visual inspection included examination of changes in level, trend, variability, immediacy of effect, and overlap across phases (Kratochwill et al., 2021). This analytic process supported determination of functional relations between the AI-supported self-coaching intervention and improvements in teacher implementation and child learning outcomes. To supplement visual analysis, effect size estimates, Tau-U (Parker et al., 2011) were calculated following completion of the study.

### 2.8. Interobserver Agreement (IOA)

IOA procedures were implemented throughout the study to ensure the reliability and accuracy of data collected on teacher implementation of EI and child responding. Two trained observers, each a graduate student in early childhood or special education, served as data collectors. A primary observer conducted all sessions, and a secondary observer independently collected reliability data for at least 30% of all observation sessions across all phases, including baseline, post-training, AI-supported self-coaching, generalization, and maintenance. Sessions selected for IOA were chosen randomly, and the primary observer was not informed which sessions would include the secondary observer, reducing the risk of observer drift or expectancy effects. Both observers were blind to the study’s hypotheses and to the specific phase of the intervention during each reliability session.

Observers completed a structured training process prior to coding study data. Training included review of the Embedded Teaching Coding Form (Rakap & Balikci, 2017), guided practice using sample observation scenarios, and calibration exercises with master-coded examples. To demonstrate readiness, observers were required to achieve at least 80% agreement with the researcher across a minimum of three practice observations before participating in live reliability sessions. Ongoing booster training sessions were provided as needed to maintain consistency in coding criteria.

During IOA sessions, both observers recorded the occurrence of complete and incomplete EI trials as well as child response categories (unprompted correct, prompted correct, incorrect, and no response) using identical definitions and data sheets. IOA was calculated using point-by-point agreement. IOA was computed using the following formula: IOA = Agreements/(Agreements + Disagreements) × 100. Agreement was scored when both observers coded the same teacher behavior (e.g., complete or incomplete EI trial) or child response category for the same opportunity. Disagreement was scored when the two observers differed in their coding for any trial or child response.

IOA data indicated that observational coding was highly reliable across dyads and phases (Table 2). During baseline, IOA ranged from 91% to 95% across the four dyads, demonstrating strong consistency in identifying naturally occurring instructional trials and child responses before the intervention was introduced. Agreement remained high in the post-training phase (92–95%) and increased slightly during the AI-supported self-coaching phase, where IOA values ranged from 95% to 97%, suggesting that observers continued to apply coding definitions consistently even as teacher and child behaviors changed. Maintenance data also showed stable reliability, with IOA ranging from 91% to 93% across dyads. All phase-specific means exceeded 90%, well above commonly accepted standards for single-case research, providing confidence that the observed changes in teacher implementation and child responding reflect true behavior change rather than measurement error.

### 2.9. Treatment Fidelity

Treatment fidelity was assessed at two levels to ensure that the intervention was implemented as designed: (a) fidelity of the researcher-delivered EI and AI-supported self-coaching training sessions and (b) fidelity of teachers’ use of the AI-supported self-coaching system. A trained graduate student attended all synchronous online EI and AI-supported self-coaching training sessions delivered via Zoom and completed a structured fidelity checklist for each session. The checklist documented whether each planned instructional activity was delivered as intended. The fidelity observer received training from the researcher prior to data collection. Following each training session, the lead trainer also completed a self-evaluation fidelity checklist to document adherence to the training plan and to note any deviations.

Fidelity of teachers’ use of the AI-supported self-coaching system was monitored through teacher self-report and research oversight. Because the research team created the individualized ChatGPT 4 accounts for each teacher, weekly engagement data including login frequency, completeness of EI trial data entry, and responses to reflection prompts were tracked. To further support fidelity, teachers completed a weekly AI Self-Coaching Fidelity Checklist, a researcher-developed tool designed to help teachers self-monitor their adherence to the self-coaching process. The checklist prompted teachers to verify completion of critical components such as (a) logging into the platform, (b) entering de-identified instructional data, (c) responding to structured reflection prompts, and (d) reviewing AI-generated feedback and recommendations. Teachers who failed to complete a weekly self-coaching cycle received a reminder email from the research team and were offered troubleshooting assistance to address potential barriers such as technical difficulties, scheduling constraints, or uncertainty about data entry procedures. Fidelity was calculated using the following standard formula: Fidelity = Number of correctly implemented components/Total number of components × 100.

Fidelity data indicated strong and consistent adherence across both the researcher-delivered training sessions and teachers’ use of the AI-supported self-coaching system. All components of the EI and AI training delivered via Zoom were implemented with 100% fidelity. Teachers’ adherence to the AI-supported self-coaching system was also high. Across the intervention, Teacher 1 demonstrated fidelity scores ranging from 90% to 100% (M = 96%), Teacher 2 from 80% to 100% (M = 94%), Teacher 3 from 90% to 100% (M = 97%), and Teacher 4 from 80% to 100% (M = 92%). Across all four teachers, self-coaching fidelity averaged 95%, with an overall range of 94% to 97%.

### 2.10. Social Validity

Social validity was assessed following the conclusion of the maintenance phase using an adapted version of the Intervention Rating Profile (IRP; Martens et al., 1985). Two parallel IRP forms were developed, one evaluating teachers’ perceptions of EI practices and the other evaluating the AI-supported self-coaching system (Rakap, 2017a). Each form consisted of 25 items, rated on a 6-point Likert-type scale ranging from 1 (strongly disagree) to 6 (strongly agree). Items addressed domains such as acceptability, feasibility, effectiveness, ease of use, and perceived sustainability of the intervention components. Both forms were administered approximately three days after teachers completed the maintenance phase, and responses were analyzed descriptively to summarize overall levels of teacher satisfaction and endorsement.

To complement the quantitative ratings, each teacher also participated in an individual semi-structured interview designed to capture more nuanced perspectives about their experiences with EI and AI-supported self-coaching. Interview questions explored teachers’ perceptions of the usefulness and relevance of the intervention, facilitators and barriers to implementation, confidence in maintaining EI practices, and suggestions for improving the training and AI system. Interviews were conducted by the researcher within one week of the final maintenance session, audio-recorded, transcribed, and analyzed using Braun and Clarke’s (2022) thematic analysis procedures to identify common themes reflecting teachers’ views on the practicality, acceptability, and long-term applicability of the intervention.

## 3. Results

**Figure 1:**
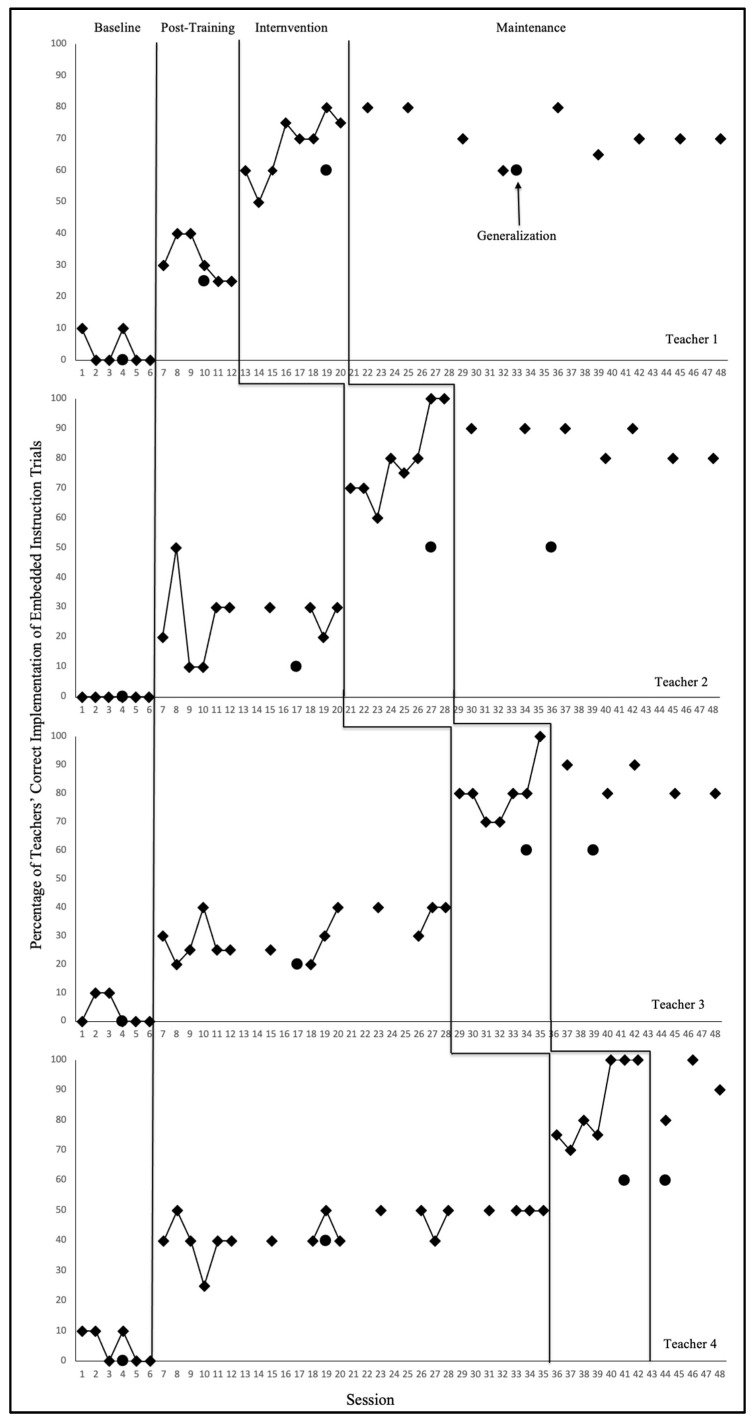
Percentage of teachers’ correct use of embedded instruction.

**Figure 2:**
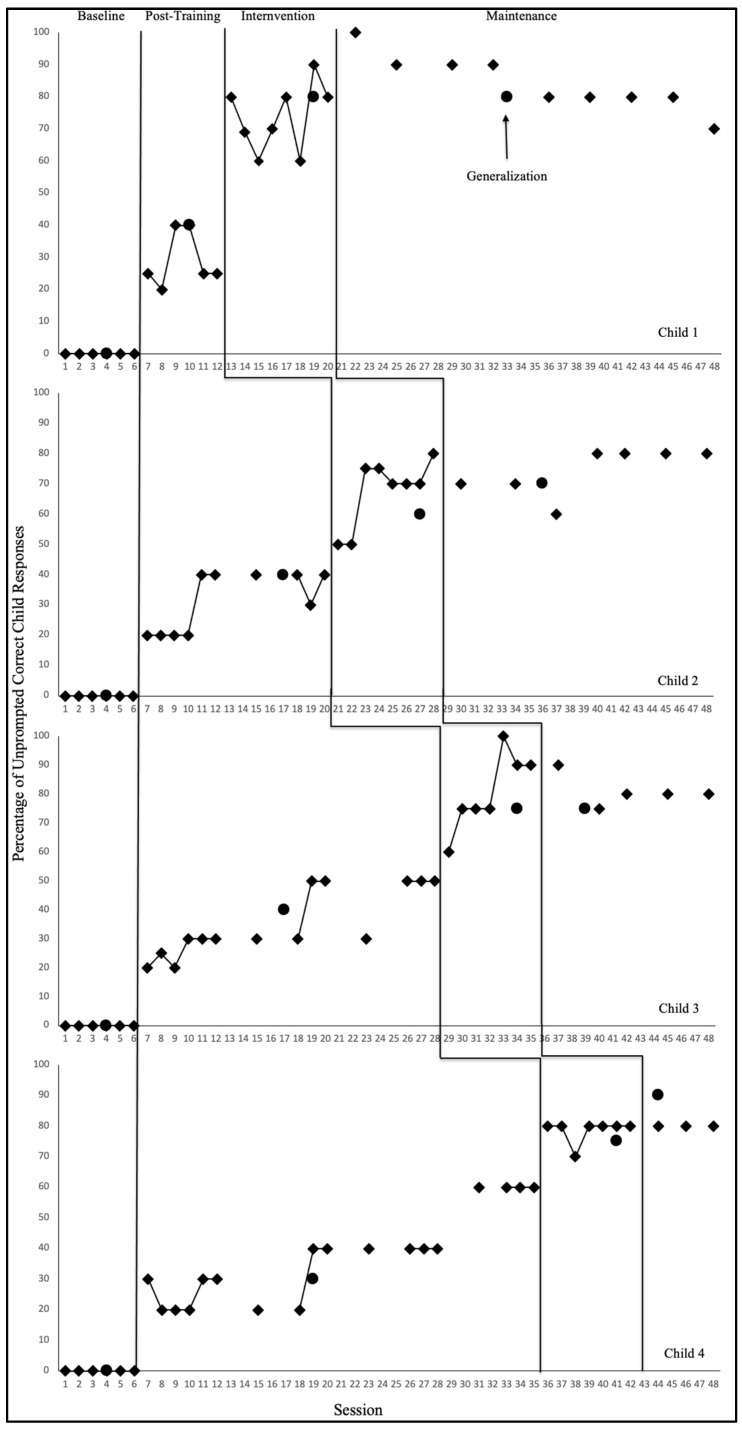
Percentage of unprompted correct child responses.

The purpose of this study was to evaluate the functional relation between AI-supported self-coaching and preschool teachers correct implementation of EI trials during naturally occurring classroom routines. In addition, the study examined the extent to which improved teacher implementation was maintained over time and generalized to non-targeted routines. Results are presented for each teacher–child dyad across all study phases, including baseline, post-training, AI-supported self-coaching, maintenance, and generalization. Phase means and performance ranges are summarized below, and complete graphed data trends are depicted in Figure 1 (teacher implementation) and Figure 2 (child learning).

### 3.1. Teachers’ Implementation of Embedded Instruction

Across all four teachers, baseline performance was uniformly low, with correct implementation ranging from 0% to 10%, indicating that teachers were not accurately delivering EI trials prior to training. Post-training performance improved for all teachers, although gains were modest and inconsistent. Experimental control was demonstrated when all four teachers showed clear, immediate, and sustained performance increases only after entering the AI-supported self-coaching phase. Maintenance and generalization data demonstrated that teachers continued to implement EI with high accuracy even after AI supports were withdrawn (see Figure 1).

#### 3.1.1. Teacher 1

Teacher 1 demonstrated minimal correct implementation during baseline (0–10%; M = 3.3%), with no evidence of an upward trend. Following EI training, her performance increased to 25–40% (M = 31.7%), indicating partial acquisition of EI procedures but with notable variability across sessions. Upon entering the AI-supported self-coaching phase, Teacher 1 showed an immediate and substantial improvement, with accuracy increasing to 50–80% (M = 67.5%). Performance demonstrated a clear accelerating trend with no overlapping data points between baseline and intervention, providing evidence of a functional relation. Teacher 1 did not meet the 80% fidelity criterion during the self-coaching phase and therefore transitioned to maintenance at the end of the 8-week intervention period. During maintenance, she sustained high levels of correct implementation (60–80%; M = 71.7%), with stable performance and low variability. Generalization probes indicated successful transfer of EI procedures to non-target routines, with 60% accuracy observed during both the intervention and maintenance phases. Tau-U for baseline–post-training, baseline–self-coaching, and baseline–maintenance comparisons was 1.00, indicating strong experimental effect (Rakap, 2015).

#### 3.1.2. Teacher 2

Teacher 2 implemented no correct EI trials during baseline (0%; M = 0%) and demonstrated no evidence of natural improvement. Following EI training, her performance increased moderately to 20–50% (M = 26%), indicating partial acquisition of EI procedures but insufficient consistency to support accurate implementation. Upon entering the AI-supported self-coaching phase, Teacher 2 exhibited an immediate and substantial improvement, with correct implementation increasing to 70–100% (M = 79.4%). The strong accelerating trend and absence of overlapping data points between baseline and intervention phases provide evidence of a functional relation. Teacher 2 met the fidelity criterion after eight weeks of intervention. During maintenance, she continued to perform at high levels (80–90%; M = 85.7%) with minimal variability. Generalization probes showed that Teacher 2 implemented EI with 60% accuracy during both the intervention and maintenance phases, suggesting successful transfer of skills to non-target routines. Tau-U for baseline–post-training, baseline–self-coaching, and baseline–maintenance comparisons was 1.00, indicating strong non-overlap and experimental effect across phases (Rakap, 2015).

#### 3.1.3. Teacher 3

Teacher 3 demonstrated minimal correct implementation during baseline (0–10%; M = 3.3%) with no evidence of an upward trend. Following EI training, performance improved modestly to 20–40% (M = 30.7%), though substantial variability within the phase suggested that training alone was insufficient to support consistent implementation. With the introduction of the AI-supported self-coaching phase, Teacher 3’s correct implementation increased immediately to 70–100% (M = 80%), showing a clear accelerating trend and no overlapping data points with baseline. Teacher 3 reached the fidelity criterion within seven weeks. During maintenance, she continued to demonstrate high and stable performance (80–90%; M = 84%). Generalization probes indicated that Teacher 3 implemented EI with 60% accuracy during both intervention and maintenance phases, confirming that EI strategies generalized beyond the originally targeted routines. Tau-U for baseline–post-training, baseline–self-coaching, and baseline–maintenance comparisons was 1.00, providing evidence of strong non-overlap and a robust experimental effect across phases (Rakap, 2015).

#### 3.1.4. Teacher 4

Teacher 4 demonstrated variable but consistently low correct implementation during baseline (0–10%; M = 5%). Following EI training, performance increased modestly to 25–50% (M = 44.2%), with fluctuations in the earlier post-training sessions and more stable data emerging in the sessions immediately preceding the intervention. Once the AI-supported self-coaching phase began, Teacher 4 exhibited a clear and immediate level change, with accuracy improving to 75–100% (M = 85.7%). No overlap was observed between baseline and self-coaching data, indicating a strong intervention effect. Teacher 4 reached criterion after seven weeks and maintained high fidelity during the maintenance phase (80–100%; M = 90%). Generalization probes showed that Teacher 4 implemented EI with 60% accuracy during both intervention and maintenance phases, confirming successful generalization of EI strategies to non-targeted routines. Tau-U comparisons across baseline–post-training, baseline–self-coaching, and baseline–maintenance were all 1.00, indicating complete non-overlap and strong evidence of a functional relation (Rakap, 2015).

#### 3.1.5. Teacher Time Commitment

To support replication and feasibility considerations, the overall time commitment required of teacher participants was documented. Across the study, teachers participated in approximately 6 h of professional development, including 4 h of embedded instruction training and 2 h of individualized training on the AI-supported self-coaching platform. During the AI-supported self-coaching phase, teachers spent an estimated 20–30 min per week engaging in planning, data entry, and reflection activities. Across all study phases, the total estimated teacher time commitment was approximately 10–12 h.

### 3.2. Child Learning Outcomes

Consistent with patterns observed in teacher implementation, all four children demonstrated clear and systematic increases in unprompted correct responding only after their teachers began implementing EI with higher accuracy. Across dyads, child performance remained at or near zero during baseline, increased modestly following EI training, and showed substantial improvements during the AI-supported self-coaching phase. These patterns parallel the functional relation demonstrated in the teacher data, suggesting that improved teacher implementation produced corresponding gains in child learning. Child outcomes for each dyad are summarized below (see Figure 2).

#### 3.2.1. Child 1

Child 1 showed no unprompted correct responses during baseline (0%; range = 0–0%). During the post-training phase, correct responding increased modestly and inconsistently to 20–40% (M = 29.2%), mirroring Teacher 1’s partial acquisition of EI procedures. Once Teacher 1 entered the AI-supported self-coaching phase and her fidelity increased substantially, Child 1 demonstrated a marked and immediate improvement in correct responding, rising to 60–90% (M = 73.6%) with a stable upward trend. Maintenance data indicated continued high performance (80–100%; M = 84.4%) even after AI support was withdrawn. Generalization probes showed that Child 1 performed with 80% accuracy during intervention and maintenance phases, demonstrating transfer of skills across untaught routines. Tau-U estimates were 1.00 for all baseline comparisons.

#### 3.2.2. Child 2

Child 2 demonstrated no correct responding during baseline (0%; M = 0%). After teacher training, correct responding increased to 20–40% (M = 31%), though data remained variable and did not indicate mastery. With the onset of Teacher 2’s AI-supported self-coaching phase, Child 2’s performance rose sharply, reaching 50–80% (M = 67.5%) and displaying a clear accelerating trend. Correct responding remained relatively high during maintenance (60–80%; M = 74.3%). In terms of generalization, Child 2 performed with 60% and 70% accuracy, respectively, during intervention and maintenance phases, indicating generalization of learned behavior to new settings. Tau-U estimates were 1.00 for all baseline comparisons.

#### 3.2.3. Child 3

Child 3 showed no unprompted correct responses during baseline (0%; M = 0%). During post-training, correct responding increased to 20–50% (M = 35.4%), paralleling Teacher 3’s post-training improvements. After entry into the self-coaching phase, Child 3 demonstrated substantial gains, with correct responding rising to 60–100% (M = 80.7%) and showing strong stability across sessions. Child 3 maintained high performance during the maintenance phase (60–100%; M = 81%) despite the removal of AI-supported teacher feedback. Generalization probes showed that Child 3 performed with 75% accuracy during intervention and maintenance phases, demonstrating transfer of skills across untaught routines. Tau-U values were 1.00 for all baseline–intervention comparisons.

#### 3.2.4. Child 4

Child 4 exhibited no unprompted correct responding during baseline (0%; M = 0%). Post-training responses increased modestly to 20–60% (M = 37.2%), consistent with the partial effects of teacher training observed for Teacher 4. When Teacher 4 entered the AI-supported self-coaching phase, Child 4’s performance increased to 70–80% (M = 78.6%) with a clear upward shift and no overlapping data points across baseline and intervention. Maintenance performance remained high (80%) despite removal of AI feedback. In terms of generalization, Child 4 performed with 75% and 90% accuracy, respectively, during intervention and maintenance phases, reflecting consistent transfer of skills across routines. Tau-U was 1.00 for all baseline–intervention comparisons, indicating a strong functional relation between teacher EI implementation and child responding.

### 3.3. Social Validity

Results from the social validity measures indicated strong teacher endorsement of both the EI practices and the AI-supported self-coaching system. Mean ratings across the four teachers on the 25-item IRP scales demonstrated high acceptability, feasibility, and perceived effectiveness of the intervention. For the EI IRP, teachers provided an average rating of 5.68 (range = 4.80–6.00), indicating that they viewed EI as highly appropriate, beneficial for their target children, and feasible to sustain during daily routines. Ratings for the AI-supported self-coaching IRP were similarly positive, with a mean score of 5.49 (range = 4.30–6.00), suggesting that teachers found the AI tool understandable, user-friendly, and helpful in supporting accurate implementation of EI. Across both measures, no teacher endorsed ratings in the “disagree” range, and all four teachers indicated that they would recommend the approach to colleagues.

Findings from the semi-structured interviews supported the quantitative results and provided deeper insight into teachers’ experiences. A major theme centered on enhanced instructional confidence, with teachers reporting that the combination of EI training and AI-guided feedback helped them feel more capable of meeting the needs of children with disabilities. One teacher explained, “*Having the AI check my data each week made me pay closer attention to how well I was teaching. I felt supported, I always had guidance waiting for me.*” Another teacher emphasized that the structured prompts kept her focused on instructional priorities, noting, “*It reminded me of questions I should ask myself as a teacher, the kind we don’t always stop to think about when the day gets busy.*” A second theme involved teachers’ perceptions of the practicality and usefulness of the weekly self-coaching cycles. Teachers appreciated the flexibility of entering data on their own time and the clarity of the graphical summaries. For example, one teacher stated, “*Seeing the graphs every Friday helped me understand exactly where my child was improving and where I needed to adjust. It felt like having a data coach.*” Others highlighted that the AI feedback reduced guesswork, describing the tool as “*clear, specific, and focused on what mattered.*”

Teachers also commented on the sustainability of the approach. All four indicated that they intended to continue using EI strategies beyond the study period, and two expressed interest in applying the AI-supported cycle with additional children in their classrooms. One teacher shared, “*Now that I know how to do this on my own, I feel like I can keep it up even without someone watching over me.*” Another remarked that the process helped her develop routines that were “*easy to keep doing because they fit into what we already do every day.*” Despite overall positive perceptions, teachers noted several practical challenges. One teacher mentioned difficulty finding time for data entry during weeks with staffing shortages. Another expressed initial uncertainty about using AI technology, although she reported that this concern diminished once she became familiar with the platform. When asked about limitations, one teacher noted that self-coaching would be more difficult to sustain if working with multiple children simultaneously, echoing concerns documented in other early childhood implementation research. Importantly, all teachers emphasized that these challenges did not diminish the overall value of the intervention.

## 4. Discussion

The purpose of this study was to evaluate whether an AI-supported self-coaching system could improve preschool teachers’ fidelity of EI implementation and, in turn, support better learning outcomes for autistic preschoolers in inclusive classrooms. The study also examined teachers’ ability to maintain and generalize EI use following the intervention and explored their perceptions of the usability and acceptability of both EI and AI-supported self-coaching. Across all four teacher–child dyads, findings demonstrated strong functional relations between the introduction of the AI-supported self-coaching system and increased teacher fidelity, with parallel improvements in children’s unprompted correct responding, and positive teacher perceptions of the intervention’s feasibility and value. These results contribute to the emerging literature on technology-enabled professional development and reaffirm the importance of ongoing implementation support for sustaining evidence-based practice in early childhood settings.

### 4.1. AI-Supported Coaching as a Mechanism for Improving EI Fidelity

Consistent with decades of PD research, teachers in this study made only modest gains following training and did not achieve high, stable fidelity until they entered the AI-supported self-coaching phase. This pattern mirrors earlier work demonstrating that workshop-only PD while useful for introducing foundational concepts, rarely produces robust or sustained changes in classroom practice (Kretlow & Bartholomew, 2010; Snyder et al., 2018). Even when the training was interactive and high quality, as in the present study, teachers struggled to translate conceptual learning into consistent practice without ongoing performance feedback. These findings align closely with previous research on EI, which similarly reported that fidelity improved minimally after training but reached criterion only with added coaching support (Rakap et al., 2024; Snyder et al., 2018).

The AI-supported self-coaching system appeared to replicate core mechanisms of coaching (goal setting, data-based reflection, and individualized feedback) without the resource demands of a live coach. Immediately upon entering this phase, all teachers demonstrated clear improvements in accuracy, strong accelerating trends, and non-overlapping data with baseline or post-training sessions. These fidelity gains were achieved in a relatively short time frame, with teachers meeting criterion within 7–8 weeks on average. Similar to findings from naturalistic instruction and other systematic teaching approaches (e.g., Barton et al., 2018; Koegel et al., 2012), improvements were rapid and sustained once accurate implementation practices were reinforced regularly. The efficiency of this model is particularly notable given that no live coaching was provided; the AI-generated summaries and recommendations appeared sufficiently precise to guide teachers’ instructional adjustments week to week.

A noteworthy pattern was that teachers with longer post-training phases did not require additional self-coaching sessions to reach fidelity. This contrasts with earlier concerns that delays between training and implementation support may slow skill acquisition or increase the likelihood that teachers revert to pre-training habits (Snyder & Wolfe, 2008). Rather than showing deterioration, teachers appeared able to maintain the skills gained during training until the self-coaching phase began. This suggests that the explicit, step-by-step structure of EI training may have provided a strong conceptual anchor that teachers could draw on later, even in the absence of immediate coaching. Programs that struggle to provide prompt coaching due to staffing or scheduling constraints may therefore still benefit from high-quality, well-structured training paired with delayed, but systematic, AI-supported coaching.

### 4.2. Maintenance and Generalization of Embedded Instruction

All teachers maintained high-fidelity implementation after withdrawal of AI supports, and they successfully generalized EI use to new routines that were not targeted during intervention. Maintenance of newly learned practices is a noted challenge in early childhood PD (Snyder et al., 2018), and the durability observed in this study suggests that AI-supported reflection cycles helped teachers internalize core decision-making processes required for sustained implementation. Teachers may have developed a stronger sense of ownership over EI procedures because self-coaching required them (not an external coach) to analyze their data, identify needs, and plan instructional adjustments. This aligns with conceptualizations of self-coaching as a way to promote metacognitive awareness and long-term skill retention when teachers are provided with the right tools and structures (Shannon et al., 2015).

Similarly, generalization to non-targeted routines is a critical marker of practice adoption. Teachers used EI with high accuracy in novel routines, suggesting that they were not simply replicating specific examples viewed during training but were instead applying EI principles flexibly across classroom contexts. This finding aligns with prior research demonstrating that when EI is implemented with fidelity, teachers can extend learning opportunities across a wide range of activities (Rakap & Parlak-Rakap, 2011; Gulboy et al., 2023). Generalization across routines is particularly important in early childhood contexts, where activities change throughout the day and where instructional opportunities arise spontaneously. The ability to adapt EI flexibly across routines indicates deeper learning and skill integration.

### 4.3. Child Learning 

Child outcomes closely paralleled teacher implementation fidelity, a finding that aligns with the EI literature and naturalistic developmental–behavioral frameworks more broadly. Across all four dyads, children demonstrated minimal gains during baseline and modest but inconsistent improvements following teacher training. In contrast, once teachers entered the AI-supported self-coaching phase and used EI with higher accuracy, each child showed clear increases in unprompted correct responding, stable upward trends, and strong maintenance effects. These results support prior evidence indicating that systematically embedded learning opportunities are highly effective for young children with autism (Snyder et al., 2015; Rakap & Balikci, 2025). Importantly, children also generalized their target skills to untaught routines. This is consistent with scholarship emphasizing that when intervention is embedded in meaningful contexts, skills are more likely to persist and transfer (Brown & Odom, 1994; Chandler et al., 1992). The findings here reinforce the developmental value of EI in supporting children’s participation, independence, and progress toward individualized goals. Generalization of child skills strengthens the case for EI as not only an effective teaching approach but one that supports functional, ecologically valid learning, learning that transfers to daily life.

### 4.4. Social Validity and Teacher Perceptions

Teachers rated both EI and the AI-supported self-coaching system highly in terms of acceptability, feasibility, and usefulness. Their interview responses offered deeper insight into why the intervention was well received. Teachers noted that the AI feedback was clear, actionable, and aligned with their classroom realities, qualities that are critical for sustaining any form of PD. They also emphasized feeling more confident, more reflective, and more equipped to make data-based instructional decisions, themes also found in previous studies evaluating EI coaching models (Rakap et al., 2024). Teachers’ reported concerns, primarily related to time demands, initial discomfort with technology, and the challenge of implementing EI for multiple children simultaneously, mirror common barriers in the early childhood literature (Rahn et al., 2019). However, these concerns did not detract from their overall endorsement of the system. The strong social validity observed in this study suggests that AI-supported self-coaching is not only feasible but also perceived as genuinely supportive of teacher learning and professional growth.

### 4.5. Implications for Practice

The results of this study offer several practical implications for early childhood programs working to improve EI in inclusive classrooms. First, the findings reaffirm that training alone is insufficient for achieving high-fidelity implementation. Programs should pair initial instruction with ongoing, structured implementation support, as these additional supports whether delivered by a coach or through technology-enabled systems are essential for helping teachers translate knowledge into consistent practice. Second, the success of the AI-supported self-coaching system highlights the value of individualized, data-driven feedback. Providing teachers with timely information about their own performance can help them identify challenges, make informed instructional adjustments, and develop a stronger sense of ownership over EI practices. Early childhood programs may consider incorporating similar self-reflection and data-use routines into their PD models, regardless of whether AI is used.

Third, because teachers maintained and generalized EI practices even after supports were withdrawn, PD systems should emphasize structures that build teachers’ capacity for independent reflection and problem solving. Embedding these self-directed components within PD may promote long-term sustainability and reduce reliance on ongoing external coaching. Finally, the strong gains in child learning observed alongside improved teacher fidelity highlight that PD efforts should remain explicitly tied to child goals and developmental outcomes. Providing teachers with tools and supports that help them monitor progress and make data-based decisions can enhance the quality and impact of instruction for children with autism and other developmental needs. Overall, the study suggests that AI-enabled supports can serve as a feasible and scalable complement to traditional coaching, particularly in settings where resources are limited. When thoughtfully implemented, such systems can strengthen teacher practice, support sustained use of evidence-based instruction, and contribute to more meaningful learning opportunities for young children in inclusive early childhood programs.

### 4.6. Limitations and Recommendation for Research

Several limitations should be considered when interpreting the findings of this study. First, although the multiple-probe across participants single-case design allowed for strong demonstrations of functional relations, the study included only four teacher–child dyads. While consistent effects across participants strengthen confidence in the intervention’s impact, the small sample limits generalizability. Future research should evaluate AI-supported self-coaching with larger and more diverse samples across multiple program types to examine variability in teacher response and contextual influences. Second, the study relied on researcher-created ChatGPT 4 accounts, and the AI system generated feedback based solely on teacher-entered data. Although this ensured privacy and consistency, it also meant the AI did not have access to contextual nuances that live coaches naturally incorporate. Future research should explore the feasibility, accuracy, and ethical considerations of integrating multimodal inputs (e.g., video, audio, classroom context cues) to enhance the precision of AI-generated feedback while maintaining strict data-protection safeguards.

Third, although teachers demonstrated strong maintenance and generalization, the study assessed maintenance only over a short-term period. It remains unknown whether teachers would sustain high-fidelity EI implementation over months or school years without continued AI or human coaching. Longitudinal research is needed to evaluate the durability of AI-supported coaching effects, particularly as classroom compositions, staffing, and instructional demands shift across the year. Fourth, the study focused on teachers implementing EI with a single target child. In practice, early childhood educators must address the needs of multiple children with diverse goals. Several teachers in this study noted that self-coaching might be more challenging when supporting multiple children simultaneously. Future studies should examine how AI-supported systems can be adapted to help teachers manage multiple goals, differentiate instruction, and allocate instructional opportunities equitably.

Fifth, the AI-supported self-coaching system was introduced only after teachers completed researcher-led training. Thus, the extent to which AI alone could support initial skill acquisition remains unknown. Future research might investigate alternative PD sequences (e.g., AI-first models, hybrid AI + peer coaching, AI-augmented communities of practice) to determine whether AI can effectively support both the acquisition and refinement of EI practices. Additionally, although teachers rated the system highly, the study did not formally measure AI trust, digital literacy, or perceived cognitive load, all of which may influence user engagement. Future work should incorporate validated measures of technology acceptance to better understand how teacher characteristics shape responsiveness to AI-supported PD.

Finally, child learning outcomes were positive but limited to unprompted correct responding on predefined objectives. Broader developmental indicators such as social engagement, communication spontaneity, or participation during group activities were not assessed. Future research could extend evaluation to include general developmental outcomes, parent-reported progress, or the impact of teacher fidelity on peer interactions and classroom-level participation patterns.

## 5. Conclusions

This study provides initial evidence that AI-supported self-coaching can strengthen preschool teachers’ use of EI and positively influence the learning of autistic children in inclusive settings. Teachers who engaged with the AI-guided planning, reflection, and feedback cycles quickly improved their instructional fidelity, sustained these gains after supports were removed, and generalized practices to new routines. Children demonstrated parallel improvements in their unprompted correct responding, underscoring the importance of high-fidelity EI for promoting meaningful learning. Teachers also viewed the AI system as practical, supportive, and easy to integrate into their weekly routines, suggesting that technology-enabled tools can reduce barriers associated with traditional coaching. While additional research is needed to evaluate long-term sustainability, use with larger and more diverse samples, and applicability when supporting multiple children, the present findings highlight the promise of AI-supported self-coaching as a scalable and accessible professional development approach for inclusive early childhood education.

## Figures and Tables

**Table 2:** Interobserver Agreement (Percent) Across Dyads and Study Phases.

Phase	Dyad 1	Dyad 2	Dyad 3	Dyad 4
Baseline	92 (88–95)	96 (92–99)	94 (90–97)	95 (90–98)
Post-Training	93 (90–97)	92 (89–94)	91 (87–95)	90 (86–94)
Self-Coaching	95 (92–98)	97 (94–99)	93 (90–96)	94 (90–97)
Maintenance	91 (87–94)	90 (85–94)	92 (88–95)	91 (87–94)

Note. Values represent mean IOA percentages with observed ranges in parentheses. All IOA values exceeded 90% indicating strong reliability across observers and study phases.

## Data Availability

The raw data supporting the conclusions of this article will be made available by the author upon request.
